# Effect of Using Different Chemical Dispersing Agents in Grain Size Analyses of Fluvial Sediments via Laser Diffraction Spectrometry

**DOI:** 10.3390/mps4030044

**Published:** 2021-06-29

**Authors:** Mubarak Abdulkarim, Haruna M. Grema, Ibrahim H. Adamu, Daniela Mueller, Melanie Schulz, Marius Ulbrich, Johannes M. Miocic, Frank Preusser

**Affiliations:** 1 Institute of Earth and Environmental Sciences, University of Freiburg, 79104 Freiburg, Germany; daniela.mueller@geologie.uni-freiburg.de (D.M.); m.schulz93@gmx.net (M.S.); Eisen01@gmx.net (M.U.); frank.preusser@geologie.uni-freiburg.de (F.P.); 2 Department of Geology, Federal University Birnin Kebbi, Kalgo 862104, Nigeria; 3 GFZ German Research Centre for Geosciences, 14473 Potsdam, Germany; hgrema@gfz-potsdam.de; 4 Department of Geology, Faculty of Physical and Computing Science, Usmanu Danfodiyo University, Sokoto 840232, Nigeria; habib.ibrahim@udusok.edu.ng; 5 Energy and Sustainability Research Institute Groningen (ESRIG), University of Groningen, 9747 AG Groningen, The Netherlands; j.m.miocic@rug.nl

**Keywords:** grain size analyses, laser diffraction spectrometry, fluvial sediments, Upper Rhine, Quaternary, chemical dispersants, flocculation

## Abstract

Laser diffraction spectrometry allows for efficiently obtaining high-resolution grain size data. However, pretreatment and dispersion of aggregates in sediment samples are essential pre-requisites for acquiring accurate results using this method. This study evaluates the effectiveness of five dispersing agents in deflocculating the investigated fluvial sediments and the resulting grain size distribution obtained by laser diffraction spectrometry. We also examine the ability of the different dispersing agents to deflocculate sediment samples treated by thermal combustion. Distilled water presented a low efficiency in deflocculating the samples and yielded a near-zero clay content for samples with an expected clay content. The other chemical dispersants were effective in dispersing aggregates and yielding clay, albeit with different efficiencies. Calgon had the highest dispersing ability, followed closely by sodium tripolyphosphate. The performance of chemical treatment with sodium oxalate approaches that of sodium tripolyphosphate. However, it leads to the formation of precipitates in the samples, obscuring the actual grain size data. Sodium pyrophosphate derived the least amount of deflocculation among the four chemical dispersants. Furthermore, all the chemical dispersants were found to be ineffective in dispersing aggregates in samples treated by thermal combustion.

## 1. Introduction

Grain size is one of the most important physical properties of sediments. The grain size distribution observed in a sediment is determined by the availability, entrainment, transport, and deposition of detrital debris [1] and thus provides valuable information regarding provenance, transport processes, and depositional mechanisms [1,2]. Systematic analysis of sediment grain size properties has been applied in various studies for the interpretation and deeper understanding of geological and geomorphological processes in several sedimentary environments, e.g., [3,4,5,6,7,8,9,10]. In addition, grain size data is relevant to applied engineering and applied geosciences; for example, when estimating load-bearing capacity and porosity. For accurate and reliable interpretations, it is imperative to obtain high-resolution grain size data.

Several analytical techniques are available for the measurement of particle size distribution and include traditional methods such as sieving, hydrometer methods, and pipette analysis. More recently, laser diffraction analysis, x-ray granulometry, and the Coulter counter method have become available [11,12]. Over the last few decades, the introduction of laser diffraction spectrometry (LDS) has provided a means to obtain high-resolution grain size data. Through laser diffraction, particle size distributions are determined by measuring the angle and intensity of scattered light from a particulate sample illuminated by a laser beam. The scattered light data is converted into particle size information using the Mie theory of light scattering or the Fraunhofer diffraction theory. This analytical technique offers extensive measurement capabilities, allowing for rapid measurement of grain size distribution over a wide range of materials, from clay to very coarse sand (0.01–3500 µm) [13]. It is thus a powerful tool for measuring grain sizes as found in many depositional environments.

However, one significant limitation to robust and reliable measurements using the LDS technique is the aggregation of particles in the suspension media [14,15,16,17]. Aggregation results from fine particles adhering to each other and forming coarser composite particles known as agglomerates or flocs. This process of flocculation is particularly problematic for particle size determination because the particle size analyzer will register the flocs as one large primary particle and not the individual grains. This often leads to an underestimation of fine particles (clay and fine silt) with an apparent corresponding overestimation of larger grain sizes. 

Another limitation of the LDS method is the presence of organic matter in samples. Organic matter impedes dispersion by binding finer-grained particles and is also problematic for measurements, as particle size analyzers cannot differentiate between the mineral components and organic material [15,18]. Thus, the removal of organic material in sediment samples is an essential prerequisite for complete dispersion and accurate particle size measurements. A widely used approach for removing organic matter is by chemical degradation using 20–30% hydrogen peroxide. This method is highly effective but time consuming, with degradation periods that can last up to several days. For this reason, some protocols adopt thermal combustion to remove organic material prior to grain size measurements, e.g., [19]. Thermal combustion offers a relatively quick and effective means to remove organic matter, but past studies have shown that it can lead to the aggregation of fine grains [15,18], thus generating biased results from grain size analysis.

To overcome the formation of flocs and improve the accuracy of measurements, it is necessary to pretreat and disperse sediment samples into primary particles before measuring grain size distributions. This is usually achieved by adding a chemical dispersing agent in the suspension medium (sediment–water mixture) [14,15,16,17]. A wide variety of chemical dispersants or deflocculating agents have been used in different studies, including sodium tripolyphosphate [20,21], sodium oxalate [20,21,22], Calgon (sodium hexametaphosphate) solution [14,17,20,21,22,23], sodium hydroxide [20,23], sodium silicate [20,21,22], and sodium pyrophosphate [16,20,21,23]. Several of these studies have investigated the effectiveness of these different chemical dispersing agents to determine the most suitable dispersant to be used for particle size measurements, e.g., [20,21,22,23]. However, their results are inconsistent. The effectiveness of these chemical dispersants varies among different soils and sediment types, and the environmental settings from which they were collected. Accordingly, selecting chemical dispersants for sample pretreatment should be based on sediment type and depositional environment. 

This study evaluates the effectiveness of different chemical dispersing agents on the grain size distribution of Quaternary fluvial sediments from the Upper Rhine Plain, France. Two experimental setups were designed: (1) The first experiment evaluated the effectiveness of four chemical dispersing agents on a range of fluvial sediments. (2) The second experiment examined the efficiency of the chemical dispersants in deflocculating sediment samples treated by thermal combustion. 

## 2. Materials and Methods

### 2.1. Samples

**Figure 1:**
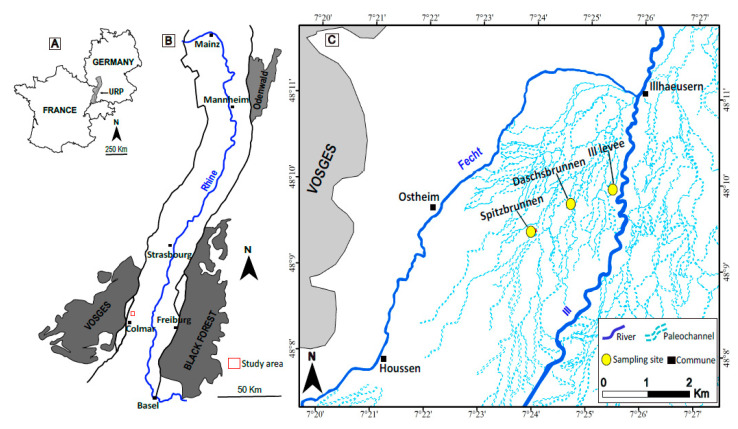
Maps showing (**A**) the location of the Upper Rhine Plain in Europe, (**B**) the location of the study area within the Upper Rhine Plain, and (**C**) the location of sample sites.

Thirty-five fluvial sediment samples were collected from three locations in the Alsatian Upper Rhine Plain, Northeastern France (Figure 1). Thirty-three of the sediment samples were obtained from two 1.7m-long cores drilled in two paleochannels, Daschsbrunnen (DB) and Spitzbrunnen (SB; Figure 1C). These paleochannels are remnants of the Late Glacial braided system of the River Rhine and are filled with fine-to coarse-grained, stratified clastic sediments from different source areas [24]. Two additional organic-rich sediment samples were taken from a levee of the adjacent Ill River (Figure 1C) at depths of 40 (IL 1) and 60 cm (IL 2), using a hand auger drill.

**Figure 2:**
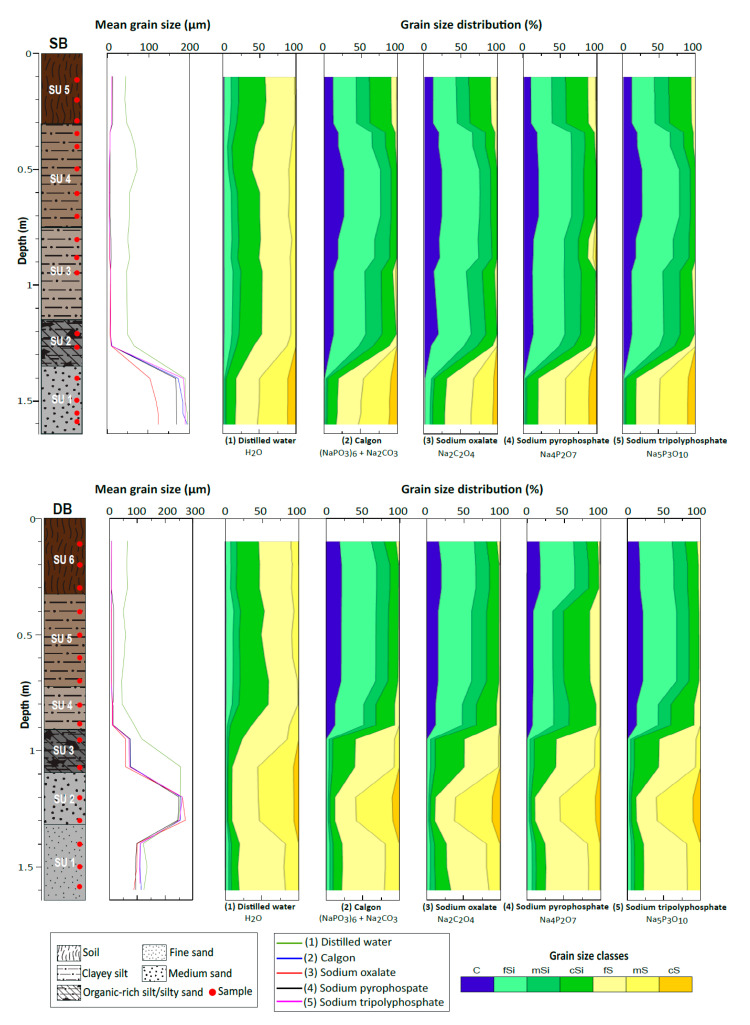
Lithologic profiles, mean grain size, and granulometric fractions of Spitzbrunnen (SB) and Daschsbrunnen (DB) sediment cores. The grain size data represent the results of measurements using five different dispersants. Grain size classes are based on the modified Udden–Wentworth scale. (C: clay; fSi: fine silt; mSi: medium silt; cSi: coarse silt; fS: fine sand; mS: medium sand; cS: coarse sand.).

The two paleochannels (DB, SB) show a general fining-upward sequence (Figure 2), typical for alluvial sequences [25]. Macroscopic descriptions of the sediment cores indicate sandy deposits at the basal parts overlain by a heterogeneous succession of fine materials (silt, clay, loam). The basal part of the SB sediment core (165–135 cm) consists of a grey fine to medium sand layer (SU-1), overlain by an organic-rich silt unit (SU-2) from 135 to 115 cm. From 115 to 75 cm, the grey clayey silt layer (SU-3) gradually grades into brownish-grey clayey silt (SU-4) from 75 to 32 cm. The uppermost part of the SB core is a dark brown silty loam (SU-5) from 32 cm to the surface. For DB, the lowermost part of the core (165–133 cm) comprises grey fine sand (SU-1), which transitions into grey medium sand (SU-2) between133 and109 cm. A dark grey silty sand unit with abundant organic fragments (SU-3) is observed from a depth of 109 to 91 cm within the core. This unit is overlain by grey clayey silt (SU-4) from 91 to 73 cm, beige to grey clayey silt (SU-5) from 73 to 32 cm, and is capped by a brown to dark brown silt loam horizon (SU-6) from 32 to 0 cm. 

Samples from the levee of the Ill River (IL 1 and IL 2) consist of dark brown to black organic-rich sandy silt, with organic content up to 20%. The sediments contain abundant organic fragments and show traces of soil formation.

### 2.2. Sample Preparation

#### 2.2.1. Experiment 1

Sediment core samples were taken from SB (*n* = 16) and DB (*n* = 17) at 10 cm intervals (one sample every 10 cm), thereby collecting material from each sediment layer. These samples were selected for experiment 1 since they consist of a wide range of fluvial sediments representing different geological source areas and depositional environments [24]. The samples were oven-dried at 35 °C for seven days, carefully homogenized, and sieved through a 2 mm sieve. The <2 mm fractions were treated with 30% hydrogen peroxide (H_2_O_2_) at 70 °C for 12 h to remove organic matter. The H_2_O_2_ treatment was repeated two to three times until the samples were completely bleached and all organic matter was degraded. Subsequently, the samples were washed with distilled water. Each sample was evenly divided into five aliquots (approximately 5 g each) as preparation for dispersion (deflocculating).

Four chemical dispersing agents and (1) distilled water were considered. The dispersants used are (2) Calgon (a mix of sodium hexametaphosphate (NaPO_3_)_6_ and sodium carbonate (Na_2_CO_3_)), (3) sodium oxalate (Na_2_C_2_O_4_), (4) sodium pyrophosphate (Na_4_P_2_O_7_), and (5) sodium tripolyphosphate (Na_5_P_3_O_10_). Distilled water was selected for this experiment to examine if water alone could deflocculate the samples without adding chemical dispersing agents. The concentration of dispersants is shown in Table 1. To each aliquot, 20 mL of a different dispersing agent was added, with one aliquot only having distilled water (20 mL) added to it. All aliquots were allowed to sit for 24 h and then were transferred to an ultrasonic bath unit (Bandelin Sonorex RK 510 H) for sonication. The samples were sonicated for 3 min on maximum sonication power. This process was performed just before measurement in the LDS system.

#### 2.2.2. Experiment 2

Sediment samples taken from the levee of the Ill River (IL 1 and IL 2) were used for this experiment as these are rich in organic matter. The bulk samples were dried in an oven at 35 °C for seven days and sieved through a 2 mm mesh. After sieving, each sample was divided into two aliquots and labeled ‘A’ and ‘B.’ To remove organic matter, the aliquots labeled ‘A’ were treated with 30% H_2_O_2_ initially at room temperature, and then at 70 °C for 12 h. In contrast, those labeled ‘B’ were heated in a muffle oven at 550 °C for 4 h [26]. After heating, the samples were transferred to a desiccator and left to cool. 

To disperse the samples, the same chemical dispersants used in experiment 1 were employed. However, distilled water was not considered as the outcome of the first experiment showed it is ineffective (see Section 3.1 and Section 4.1). Both sets of samples were divided into aliquots, and 20 mL of the four different dispersants were added and allowed to sit for 24 h. Subsequently, they were sonicated (Bandelin Sonorex RK 510 H) for 3 min just before measurement with the LDS system. 

### 2.3. Measurement Equipment and Protocols

Particle size measurements were performed using a Mastersizer 3000 laser particle size analyzer (Malvern Panalytical, Malvern, UK) equipped with an automated wet dispersion unit (Hydro EV). This setup uses water as dispersing medium and is able to measure particle size over the range of 0.01 to 3500 μm. The grain size distribution of samples was calculated using the Mie optical model [13,15]. Several test runs were performed before the measurements to determine the ideal instrumental settings and standard operating procedure for the sample analyses (Table 2).The test runs showed that using the ultrasonication function of the Mastersizer led to the formation of air bubbles, which biased the results. Furthermore, it was shown that ultrasonication can be associated with a break up of particles and immediate reaggregation of samples [14,16,27]. Thus, the inline sonication of the Mastersizer was turned off for all sample measurements.

Particle size distributions were measured using the wet dispersion unit, filled with 500 mL of clean tap water. The measurement was initialized by aligning the system and recording the background signal from the water circulating through the measurement cells and windows. This background value was later automatically subtracted from the particle size measurement results. After the background measurement, the sample was slowly added into the liquid medium (water) until the desired obscuration range was reached (Table 2). Five measurement runs of the samples were made using a 10 s measurement time (Table 2). The measurements were automatically averaged, and the mean of five measurements was selected as the final value. The measurement process was repeated three times for each aliquot to ensure stable and repeatable results.

The measurement results for each aliquot were averaged using Microsoft Excel and further analyzed with GRADISTAT statistical program v 8.0 [28] to determine grain size statistical parameters, including mean, and percentages of grain size fractions (% of sand, silt, and clay). The parameters were calculated using the statistical formulas of Folk and Ward [3], while grain size classes were categorized according to the Udden–Wentworth grain size scale [29,30].

## 3. Results

### 3.1. Experiment 1

Down core variations in grain size distribution obtained using the four different chemical dispersing agents and distilled water are presented in Figure 2 and Table 3.The complete grain size distribution data are found in the supplementary material (Tables S1–S10). For both the SB and DB cores, treatment with distilled water showed mean diameter values between 44 and 62 µm for the soil horizons, 50 and 65 µm for clayey silts, 57 µm for the silt unit of SB (SU-2), and 185 µm for the silty sand (SU-3) of DB. Treatment with distilled water yielded grain size classes dominated by coarse silt and fine sand with near-zero clay content for these stratigraphic units. By contrast, all four chemical dispersants offer mean diameter values of ~5–12 µm for the top-soil horizons, ~4–12 µm for the clayey silts, 7–8 µm for the silt (SU-2) of SB, and ~60–70 µm for the silty sand unit (SU-3) of DB. These dispersants show the dominance of clay and fine silt with additional medium and coarse silt fractions within the upper units. However, when using sodium pyrophosphate, a fine sand fraction within the clayey silt unit of the SB core between 0.7 to 0.9 m is observed.

The sandy basal units (SU-1 in SB; SU-1 and SU-2 in DB) show comparable grain size distributions with all the pretreatment methods, except for sodium oxalate, which differed considerably within the medium sand (SU-1) of the SB core. Pretreatment with sodium oxalate yielded a much lower mean diameter of 117 µm for this medium sand, while the other dispersion methods gave mean diameter values of ~170–190 µm. For the DB core, mean diameter values of ~90–125 µm were obtained for the fine sand unit (SU-1) and ~250–270 µm for the medium sand (SU-2) in DB. Overall, all five treatments indicated similar grain size classes for the lower sand units, dominated by coarse silt, fine sand, medium sand, and additional coarse sand fractions.

### 3.2. Experiment 2

**Figure 3:**
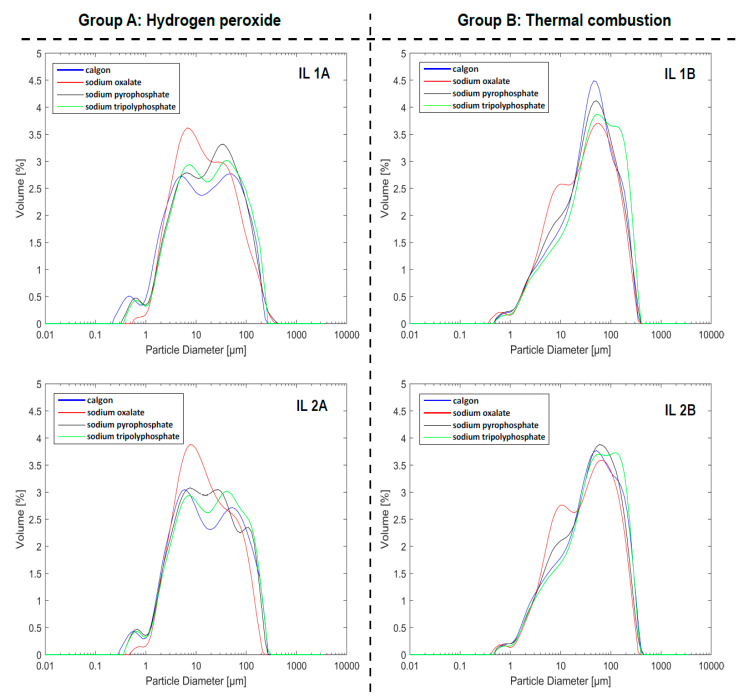
Grain size distribution curves of sediment samples IL 1 and IL 2, with Group A being treated using hydrogen peroxide and Group B with thermal combustion. Each curve represents the average of five measurements.

**Figure 4:**
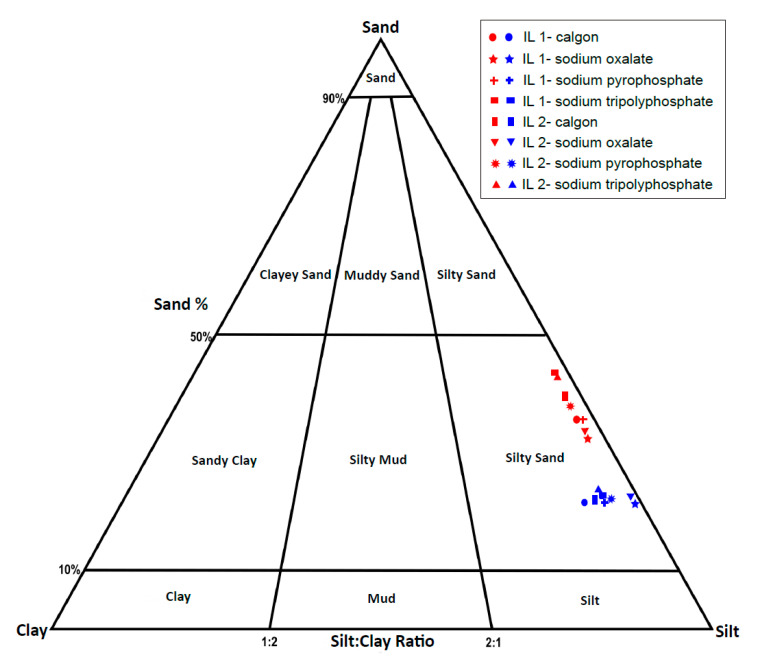
Ternary diagram illustrating the proportions of sand, silt, and clay in IL 1 and IL 2 sediment samples. The samples treated with H_2_O_2_ (Group A) are represented with blue while red represent samples treated by thermal combustion (Group B).

The results of this experiment are presented graphically as grain size distribution curves in Figure 3 and as a ternary diagram in Figure 4. Table 4 shows the statistical summary parameters of the sediments. For the complete dataset, the reader is referred to the supplementary material (Tables S11 and S12). In general, the grain size distribution curves of the samples treated with H_2_O_2_ (Group A) show asymmetrical and trimodal patterns with a wide grain size distribution (Figure 3). The curves illustrate a coarse component (peaks within very coarse silt), a fine component (peaks within fine-medium silt), and an additional fine grain size peak (minor mode) at ~0.3–0.8 μm. However, the curves of the samples dispersed with sodium oxalate after H_2_O_2_ treatment exhibit a bimodal distribution and have modes within the fine and medium silts.

A noticeable shift of the distribution curves to the coarser fractions is observed for the sediments treated by thermal combustion in Group B. These curves are characterized by different distribution patterns (unimodal, bimodal, and trimodal), negative skewness, and tails in the clay fraction. The primary modes are concentrated in the very coarse silt (~50 μm) and fine sand (~130 μm) regions. These distribution patterns indicate poor sorting of the sediments (with the exception of pretreatment with sodium tripolyphosphate) and a dominance of coarse silt and fine sand, with very little clay. Furthermore, similar to the Group A samples, the samples dispersed with sodium oxalate after combustion also show pronounced modes in the medium silt range (~10–12 μm), indicating a higher percentage of medium silt in the samples. Minimum grain sizes of ~0.21 μm were measured for Group A, while for Group B, the minimum grain size was ~0.4 μm.

The ternary plot (Figure 4) shows a similar relationship between the two sets of samples; both samples plot in the sandy silt section, but those treated by thermal combustion (Group B) show higher amounts of sand and slightly lower clay fractions. Overall, there is an apparent difference in the grain size distribution acquired through the two different pretreatment techniques. This difference is further highlighted by the statistical summary parameters in Table 4.

Table 4 presents some grain size statistical properties of the samples treated with H_2_O_2_(Group A) and thermal combustion (Group B). For samples treated with H_2_O_2_, the mean grain sizes ranged from 15 to 19 μm, while the clay fraction varied from 5.5 to 10%. These values indicate the dominance of fine to coarse silt in the samples, with a considerable amount of clay. Conversely, samples treated by thermal combustion showed much higher mean values, ranging from ~32 to 43 μm, at the same time showing decreased clay contents (2.3 to 2.8%). Coarse to very coarse silts and fine sand dominated these samples, with very little clay.

## 4. Discussion

### 4.1. Effectiveness of Dispersing Agents

The results show that the application of different dispersing agents had a significant effect on the grain size data obtained. This effect was most noticeable in the fine-grained sediments and to a lesser extent in the sandy units. Upon quantifying the clay content, it can be seen that distilled water yielded near-zero clay for all the stratigraphic units. This implies that water alone is incapable of breaking down the aggregates and dispersing the investigated sediments. Hence, the particle sizes recorded are much larger than the primary particle sizes of the sediment. However, with the application of chemical dispersants, it was observed that aggregates were efficiently removed from the samples, albeit with different efficiencies. For all tested sediments, Calgon was the most effective deflocculant, efficiently dispersing the samples and yielding the highest clay contents (24 and 21% for the two clayey silt units). This result ties well with the findings of previous studies, e.g., [14,21,22], who found Calgon to be the best dispersant for a range of soil and sediment samples and recommend its usage for granulometric studies. Chemical treatment with sodium tripolyphosphate closely matches the results of Calgon, showing very similar grain size distribution and clay content (clay fractions reach 23 and 21% in the clayey silts). It is important to highlight that sodium tripolyphosphate is not as commonly used compared to Calgon, but results from this study indicate that it was equally effective for the range of fluvial sediments we analyzed.

The results of pretreatment with sodium oxalate are similar to those of Calgon and sodium tripolyphosphate, but the apparent clay contents were reduced to 12–22% in clayey silts and 11–19% in the soil horizon. Furthermore, it was observed that within the lower sandy units, there was a considerable increase in the proportions of clay and silt with sodium oxalate pretreatment. These apparent increased clay and silt contents in the sands were most likely not related to the sediments but might be explained by very fine (silt-sized) precipitates formed by the sodium oxalate. Previous studies [15,21] noted that dispersing agents consisting of oxalates usually react with calcium to form insoluble precipitates. Bearing this in mind and evaluating the results presented here, it should be considered that treatment with sodium oxalate leads to the formation of insoluble precipitates, which are registered as clay and silt fractions by the LDS system.

Regarding the use of sodium pyrophosphate, it can be observed that this chemical dispersant showed unsatisfactory dispersion and the lowest ability to disaggregate flocs. The lowest clay contents of the four dispersants were quantified by sodium pyrophosphate (clay content varied from 8–19% in the clayey silts), highlighting its lower efficiency in dispersing the sediments analyzed. 

### 4.2. Effect of Thermal Combustion on Dispersing Agent Performance

The results of the two treatment methods show a systematic shift of the grain size distribution towards the coarse-grained fraction (very coarse silt and fine sand) when thermal combustion was applied before the addition of chemical dispersants. This increase in grain size indicates that the burning of the sediments was accompanied by the aggregation of fine grains (clay and fine silt) in the samples. This confirms previous findings [15,18], which proposed that thermal combustion leads to aggregation of samples. From the experiments conducted, it was shown that the application of chemical dispersants was effective in deflocculating aggregates in all sediment samples analyzed. However, when the samples were treated by thermal combustion, the chemical dispersants examined in this study were relatively ineffective; aggregates were not entirely broken down, and clays were underrepresented. Thus, the findings of this experiment demonstrate that while thermal combustion offers a fast and efficient method of degrading organic matter, it will most likely lead to an increase in grain size due to aggregation. Hence, it is an unsuitable pretreatment method to remove organics for grain size analysis.

The results of the second experiment further highlight the difference in the effectiveness of the four chemical dispersing agents. Analogous to the first experiment, Calgon offered the best dispersive effect for the samples from the Ill River levee, which confirms Calgon as the most effective dispersant for all the samples we analyzed. In contrast to the first experiment, where sodium tripolyphosphate showed better effectiveness than sodium pyrophosphate, it is interesting to note that the dispersing capacity of sodium pyrophosphate closely matched that of sodium tripolyphosphate in the second experiment. Furthermore, comparisons of the results show that dispersion with sodium oxalate was the least effective for the sediments from the levee of the Ill River and again was associated with the formation of insoluble silt-sized precipitates. The effectiveness of sodium oxalate is obscure since it leads to silt-sized precipitates, which can be mistaken for fine fractions within the samples analyzed. Although sodium pyrophosphate is more effective than sodium oxalate in the second set of samples, it cannot be concluded that it is generally more effective. Further tests need to be conducted to ascertain these findings.

## 5. Conclusions

The experiments conducted enable the following conclusions:

- Calgon (sodium hexametaphosphate + sodium carbonate) is the most effective dispersing agent, yielding the highest clay content.
- Sodium tripolyphosphate shows almost equal effectiveness to Calgon, albeit with small differences.
- Sodium oxalate shows similar results but will potentially lead to an increase in silt-sized particles, due to the formation of precipitates.
- Sodium pyrophosphate is the least effective of the four dispersing agents.
- Thermal combustion of sediments promotes aggregate formation, and chemical dispersants are relatively ineffective for dispersing such aggregates.
- Thermal combustion should be avoided as a pretreatment method for the determination of grain size distribution.
- The pretreatments were carried out on Quaternary fluvial sediments from the Upper Rhine Plain, but the results may also be applicable to sediments from similar depositional environments.

## Figures and Tables

**Table 1:** Details and concentration of chemical dispersing agents used.

Dispersing Agent (Deflocculant)	Concentration	Citation
(1) Distilled water	-	-
(2) Calgon ^1^ ((NaPO_3_)_6_+ Na_2_CO_3_)	33 g of sodium hexametaphosphate and 7 g of sodium carbonate per liter of distilled water	Kaur and Fanourakis [22]
(3) Sodium oxalate (Na_2_C_2_O_4_)	5 g of sodium oxalate per liter of distilled water	Kaur and Fanourakis [22]
(4) Sodium pyrophosphate (Na_4_P_2_O_7_)	44.6 g of sodium pyrophosphate per liter of distilled water	Wintermyer and Kinter [20]
(5) Sodium tripolyphosphate (Na_5_P_3_O_10_)	29.4 g of sodium tripolyphosphate per liter of distilled water	Wintermyer and Kinter [20]

^1^ Calgon solution used for this study was a mixture of 33 g sodium hexametaphosphate buffered with 7 g of sodium carbonate per liter of distilled water. This should not be confused with the water softener ‘Calgon’ with its main ingredient being polycarboxylates.

**Table 2:** Malvern Mastersizer 3000 instrument settings adopted for this study.

Parameter	Specification
Particle refractive index	1.55
Dispersant refractive index	1.33
Absorption index	0.1
Obscuration	Fine sediments: 5–10%; coarse sediments: 10–15%
Measurement duration	10 s
Measurement cycle	5
Stirrer speed	1660 rpm
Ultrasonication	Off
Size fraction bins	101 bins (0.01 to 3500 µm)

**Table 3:** Mean grain size (MGS) and clay content determined through the use of different dispersants for each stratigraphic unit (SU) of the Spitzbrunnen (SB) and Daschsbrunnen (DB) sites. The data of samples from each stratigraphic unit were averaged (arithmetic mean) and are given together with the standard deviation.

S/U	Distilled Water		NaPO_3_)_6_ + Na_2_CO_3_		Na_2_C_2_O_4_		Na_4_P_2_O_7_		Na_5_P_3_O_10_	
	MGS (µm)	Clay (%)	MGS (µm)	Clay (%)	MGS (µm)	Clay (%)	MGS (µm)	Clay (%)	MGS (µm)	Clay (%)
SB SU-5	43.9 ± 2.2	1.0 ± 0.1	9.6 ± 0.1	12.1 ± 0.1	9.8 ± 0.2	11.7 ± 0.1	11.7 ± 0.2	9.7 ± 0.2	9.6 ± 0.1	11.6 ± 0.1
SB SU-4	60.2 ± 8.5	0.6 ± 0.2	4.0 ± 1.0	23.9 ± 4.4	4.3 ± 0.7	22.0 ± 2.4	5.1 ± 0.1	19.4 ± 1.1	4.2 ± 1.0	23.0 ± 3.7
SB SU-3	49.3 ± 3.9	0.8 ± 0.1	5.4 ± 1.2	16.9 ± 3.5	5.0 ± 1.4	17.9 ± 4.5	7.4 ± 0.5	12.5 ± 0.8	5.6 ± 1.3	15.0 ± 3.3
SB SU-2	56.5 ± 11.7	0.7 ± 0.2	8.2 ± 2.4	11.0 ± 2.5	6.8 ± 2.3	13.9 ± 7.1	7.9 ± 1.8	10.9 ± 2.8	7.8 ± 1.4	10.4 ± 2.0
SB SU-1	192.7 ± 3.6	0.1 ± 0.1	183.9 ± 9.3	0.1 ± 0.1	117.3 ± 9.7	0.5 ± 0.1	168.1 ± 1.0	0.1 ± 0.1	188.6 ± 2.1	0.1 ± 0.1
DB SU-6	62.8 ± 1.6	0.4 ± 0.1	4.9 ± 0.7	20.4 ± 1.5	5.1 ± 0.5	19.0 ± 2.1	5.3 ± 0.3	18.0 ± 0.8	5.8 ± 0.4	16.7 ± 1.0
DB SU-5	49.7 ± 5.8	0.8 ± 0.1	4.7 ± 0.1	20.8 ± 0.2	5.8 ± 0.1	15.7 ± 0.1	14.2 ± 0.1	8.8 ± 0.1	4.7 ± 0.1	21.3 ± 0.1
DB SU-4	65.8 ± 30.8	0.4 ± 0.4	8.3 ± 0.1	12.3 ± 0.1	8.5 ± 0.1	12.1 ± 0.1	11.4 ± 0.1	8.0 ± 0.1	10.2 ± 0.1	12.8 ± 0.1
DB SU-3	184.9 ± 98.6	0.1 ± 0.1	74.0 ± 1.3	0.3 ± 0.1	56.2 ± 0.2	0.4 ± 0.1	73.1 ± 1.1	0.3 ± 0.1	69.4 ± 1.3	0.3 ± 0.1
DB SU-2	251.1 ± 4.0	0.1 ± 0.1	255.4 ± 2.1	0.2 ± 0.1	267.0 ± 7.7	2.3 ± 0.1	247.2 ± 2.8	0.2 ± 0.1	256.6 ± 7.0	0.2 ± 0.1
DB SU-1	125.8 ± 7.5	0.3 ± 0.1	110.6 ± 2.3	0.3 ± 0.1	94.1 ± 6.8	0.3 ± 0.1	94.3 ± 2.6	0.5 ± 0.1	108.4 ± 1.0	0.4 ± 0.1

**Table 4:** Summary of statistical grain size parameters; mean grain size (MGS), and clay content for samples IL1 and IL2, with Group A being treated using hydrogen peroxide and Group B with thermal combustion.

		(NaPO_3_)_6_+Na_2_CO_3_		Na_2_C_2_O_4_		Na_4_P_2_O_7_		Na_5_P_3_O_10_	
**Sample ID**	Treatment Method	MGS (µm)	Clay (%)	MGS (µm)	Clay (%)	MGS (µm)	Clay (%)	MGS (µm)	Clay (%)
IL 1A	Hydrogen peroxide	15.3	9.5	15.4	3.6	17.9	6.2	19.1	5.6
IL 1B	Thermal combustion	35.8	2.7	30.0	2.8	33.9	2.8	43.3	2.3
IL 2A	Hydrogen peroxide	17.1	6.6	15.5	3.2	17.6	5.9	19.6	5.5
IL 2B	Thermal combustion	38.0	2.6	31.6	2.3	35.6	2.5	41.4	2.3

## Data Availability

The data that supports the findings of this study are available in the supplementary material of this article. All other data are available from the corresponding author upon reasonable request.

## Supplementary Materials

The following are available online at https://www.mdpi.com/article/10.3390/mps4030044/s1, Table S1, Mean grain size and percentage of grain size fractions determined through the use of distilled water for the sediment samples of the Spitzbrunnen (SB) core, Table S2, Mean grain size and percentage of grain size fractions determined through the use of Calgon for the sediment samples of the Spitzbrunnen (SB) core, Table S3, Mean grain size and percentage of grain size fractions determined through the use of Sodium oxalate for the sediment samples of the Spitzbrunnen (SB) core, Table S4, Mean grain size and percentage of grain size fractions determined through the use of Sodium pyrophosphate for the sediment samples of the Spitzbrunnen (SB) core, Table S5, Mean grain size and percentage of grain size fractions determined through the use of Sodium tripolyphosphate for the sediment samples of the Spitzbrunnen (SB) core, Table S6, Mean grain size and percentage of grain size fractions determined through the use of distilled water for the sediment samples of the Daschsbrunnen (DB) core, Table S7, Mean grain size and percentage of grain size fractions determined through the use of Calgon for the sediment samples of the Daschsbrunnen (DB) core, Table S8, Mean grain size and percentage of grain size fractions determined through the use of Sodium oxalate for the sediment samples of the Daschsbrunnen (DB) core, Table S9, Mean grain size and percentage of grain size fractions determined through the use of Sodium pyrophosphate for the sediment samples of the Daschsbrunnen (DB) core, Table S10, Mean grain size and percentage of grain size fractions determined through the use of Sodium tripolyphosphate for the sediment samples of the Daschsbrunnen (DB) core, Table S11, Mean grain size and percentage of grain size fractions determined through the use of different chemical dispersing agents for IL 1A and IL 1B sediment samples, Table S12, Mean grain size and percentage of grain size fractions determined through the use of different chemical dispersing agents for IL 2A and IL 2B sediment samples.
