# Supplementary material for: Effect of Using Different Chemical Dispersing Agents in Grain Size Analyses of Fluvial Sediments via Laser Diffraction Spectrometry
Source: Methods Protoc. 2021 Jun 29;4(3):44. doi: 10.3390/mps4030044 (PMC8293417; doi:10.3390/mps4030044)
Supplement: Supplementary file 1 [file mps-04-00044-s001.zip › mps-1247331-supplementary.pdf]

Table S1. Mean grain size and percentage of grain size fractions determined through the use of distilled water for the sediment samples of the Spitzbrunnen (SB) core.

Sample ID	Depth (cm)	Mean grain size (μm)	Clay (%)	Fine Silt (%)	Medium Silt (%)	Coarse Silt (%)	Fine Sand (%)	Medium Sand (%)	Coarse Sand (%)
**SB-10-D**	10.0	43.6	1.0	9.5	10.1	36.7	40.5	2.3	0.0
**SB-20-D**	20.0	41.9	1.0	9.7	10.4	37.5	39.7	1.7	0.0
**SB-30-D**	30.0	46.3	0.8	8.3	9.0	37.2	42.8	1.7	0.0
**SB-34-D**	34.0	56.3	0.6	7.0	7.8	32.8	46.9	4.9	0.0
**SB-40-D**	40.0	66.2	0.4	5.0	6.2	31.3	50.9	6.1	0.0
**SB-50-D**	50.0	72.0	0.5	6.3	6.3	26.1	50.8	10.1	0.0
**SB-60-D**	60.0	53.2	0.8	9.9	8.6	30.7	40.8	9.2	0.0
**SB-70-D**	70.0	53.3	0.8	10.3	8.9	29.9	39.8	10.3	0.0
**SB-80-D**	80.0	48.8	0.9	10.9	9.6	29.7	43.2	5.9	0.0
**SB-88-D**	88.0	53.5	0.8	9.6	9.2	29.3	43.1	8.1	0.0
**SB-94-D**	94.0	45.7	0.9	12.2	10.8	29.4	39.2	7.4	0.0
**SB-121-D**	121.0	48.2	0.9	10.8	9.8	31.2	40.2	7.2	0.0
**SB-126-D**	126.0	64.7	0.6	9.3	8.0	23.6	46.1	12.4	0.0
**SB-140-D**	140.0	189.4	0.0	1.6	2.0	13.5	32.5	39.1	11.3
**SB-150-D**	150.0	189.9	0.0	1.6	2.0	13.5	32.3	39.3	11.3
**SB-155-D**	155.0	194.8	0.0	1.6	1.9	13.0	31.5	40.1	11.9
**SB-160-D**	160.0	196.6	0.0	1.7	2.0	12.4	30.6	41.8	11.5

Table S2. Mean grain size and percentage of grain size fractions determined through the use of Calgon for the sediment samples of the Spitzbrunnen (SB) core.

Sample ID	Depth (cm)	Mean grain size (μm)	Clay (%)	Fine Silt (%)	Medium Silt (%)	Coarse Silt (%)	Fine Sand (%)	Medium Sand (%)	Coarse Sand (%)
**SB-10-C**	10.0	9.7	12.0	31.9	18.3	29.8	8.0	0.1	0.0
**SB-20-C**	20.0	9.6	12.1	32.0	18.4	29.6	7.7	0.3	0.0
**SB-30-C**	30.0	9.4	12.2	32.1	18.6	29.6	7.2	0.2	0.0
**SB-34-C**	34.0	5.1	19.1	46.5	18.5	13.6	2.4	0.0	0.0
**SB-40-C**	40.0	5.1	19.2	46.7	18.5	13.5	2.1	0.0	0.0
**SB-50-C**	50.0	3.3	27.1	50.3	14.4	8.2	0.0	0.0	0.0
**SB-60-C**	60.0	3.3	27.1	50.3	14.5	8.1	0.0	0.0	0.0
**SB-70-C**	70.0	3.3	27.1	50.4	14.5	8.1	0.0	0.0	0.0
**SB-80-C**	80.0	4.6	18.9	50.5	20.9	9.6	0.1	0.0	0.0
**SB-88-C**	88.0	4.6	18.9	50.5	20.9	9.6	0.1	0.0	0.0
**SB-94-C**	94.0	6.8	12.9	42.9	22.5	15.9	4.0	1.8	0.1
**SB-121-C**	121.0	6.5	12.8	44.3	22.4	19.6	0.9	0.0	0.0
**SB-126-C**	126.0	9.9	9.2	38.6	19.7	22.1	10.4	0.0	0.0
**SB-140-C**	140.0	172.6	0.1	2.2	2.4	14.8	34.6	37.7	8.3
**SB-150-C**	150.0	183.8	0.0	2.0	2.2	13.7	32.6	39.3	10.2
**SB-155-C**	155.0	183.9	0.0	2.0	2.2	13.7	32.6	39.6	9.9
**SB-160-C**	160.0	195.4	0.0	1.8	2.0	12.5	30.6	41.9	11.2

Table S3. Mean grain size and percentage of grain size fractions determined through the use of Sodium oxalate for the sediment samples of the Spitzbrunnen (SB) core.

Sample ID	Depth (cm)	Mean grain size (µm)	Clay (%)	Fine Silt (%)	Medium Silt (%)	Coarse Silt (%)	Fine Sand (%)	Medium Sand (%)	Coarse Sand (%)
**SB-10-O**	10.0	10.0	11.7	31.7	17.7	29.7	9.0	0.2	0.0
**SB-20-O**	20.0	9.8	11.6	32.5	17.6	29.3	8.4	0.6	0.0
**SB-30-O**	30.0	9.5	11.7	33.4	17.6	29.3	7.6	0.5	0.0
**SB-34-O**	34.0	5.1	19.6	46.5	17.6	13.3	2.8	0.2	0.0
**SB-40-O**	40.0	5.1	19.1	47.4	17.2	12.9	2.9	0.5	0.0
**SB-50-O**	50.0	3.8	23.8	52.0	15.1	9.1	0.0	0.0	0.0
**SB-60-O**	60.0	3.8	23.7	52.6	14.8	8.8	0.0	0.0	0.0
**SB-70-O**	70.0	3.8	23.8	52.0	15.1	9.1	0.0	0.0	0.0
**SB-80-O**	80.0	4.3	20.0	53.7	18.4	7.8	0.0	0.0	0.0
**SB-88-O**	88.0	4.1	20.8	53.8	18.2	7.1	0.0	0.0	0.0
**SB-94-O**	94.0	6.6	12.7	44.8	21.9	17.0	3.6	0.0	0.0
**SB-121-O**	121.0	5.1	19.0	45.3	20.1	15.5	0.0	0.0	0.0
**SB-126-O**	126.0	8.4	8.9	45.8	18.5	20.6	6.3	0.0	0.0
**SB-140-O**	140.0	103.2	0.5	9.2	4.0	17.1	37.1	27.5	4.6
**SB-150-O**	150.0	118.7	0.5	7.5	3.6	16.9	37.0	29.5	5.1
**SB-155-O**	155.0	123.6	0.5	7.2	3.5	16.5	36.4	30.1	5.8
**SB-160-O**	160.0	123.6	0.5	7.2	3.5	16.5	36.4	30.1	5.8

Table S4. Mean grain size and percentage of grain size fractions determined through the use of Sodium pyrophosphate for the sediment samples of the Spitzbrunnen (SB) core.

Sample ID	Depth (cm)	Mean grain size (µm)	Clay (%)	Fine Silt (%)	Medium Silt (%)	Coarse Silt (%)	Fine Sand (%)	Medium Sand (%)	Coarse Sand (%)
**SB-10-P**	10.0	11.9	9.5	28.8	19.2	30.6	10.9	1.0	0.0
**SB-20-P**	20.0	11.7	9.7	28.9	19.2	30.5	10.5	1.2	0.0
**SB-30-P**	30.0	11.5	9.9	29.1	19.3	30.5	10.2	1.1	0.0
**SB-34-P**	34.0	5.3	18.1	46.9	18.6	13.5	2.9	0.0	0.0
**SB-40-P**	40.0	5.2	18.4	47.2	18.6	13.5	2.2	0.0	0.0
**SB-50-P**	50.0	5.1	20.0	45.7	16.7	17.3	0.2	0.0	0.0
**SB-60-P**	60.0	5.1	20.2	45.6	16.7	17.2	0.3	0.0	0.0
**SB-70-P**	70.0	5.1	20.4	45.5	16.7	17.1	0.4	0.0	0.0
**SB-80-P**	80.0	7.5	13.2	42.6	19.4	13.8	7.8	2.7	0.6
**SB-88-P**	88.0	7.8	12.7	43.3	18.7	13.2	7.2	3.6	1.3
**SB-94-P**	94.0	6.8	11.6	43.9	23.4	20.1	0.9	0.1	0.0
**SB-121-P**	121.0	6.7	12.9	43.7	21.5	19.4	2.5	0.0	0.0
**SB-126-P**	126.0	9.2	9.0	40.1	20.3	22.5	8.2	0.0	0.0
**SB-140-P**	140.0	166.6	0.0	1.6	2.1	16.0	37.8	32.7	9.8
**SB-150-P**	150.0	168.1	0.0	1.8	2.1	15.7	37.5	32.2	10.6
**SB-155-P**	155.0	168.8	0.0	1.7	2.1	15.7	37.5	32.8	10.3
**SB-160-P**	160.0	168.8	0.0	1.7	2.1	15.7	37.5	32.8	10.3

Table S5. Mean grain size and percentage of grain size fractions determined through the use of Sodium tripolyphosphate for the sediment samples of the Spitzbrunnen (SB) core.

Sample ID	Depth (cm)	Mean grain size (μm)	Clay (%)	Fine Silt (%)	Medium Silt (%)	Coarse Silt (%)	Fine Sand (%)	Medium Sand (%)	Coarse Sand (%)
**SB-10-T**	10.0	9.7	11.5	31.4	19.7	30.3	6.7	0.4	0.0
**SB-20-T**	20.0	9.6	11.6	31.6	19.8	30.4	6.5	0.3	0.0
**SB-30-T**	30.0	9.4	11.7	31.8	19.9	30.5	6.0	0.1	0.0
**SB-34-T**	34.0	5.3	18.9	45.6	18.4	13.4	3.6	0.2	0.0
**SB-40-T**	40.0	5.2	19.0	45.7	18.4	13.4	3.5	0.1	0.0
**SB-50-T**	50.0	3.5	25.6	51.6	14.8	8.0	0.0	0.0	0.0
**SB-60-T**	60.0	3.5	25.7	51.6	14.9	7.8	0.0	0.0	0.0
**SB-70-T**	70.0	3.4	25.7	51.7	14.9	7.7	0.0	0.0	0.0
**SB-80-T**	80.0	4.9	16.8	51.7	21.8	9.6	0.0	0.0	0.0
**SB-88-T**	88.0	4.9	16.9	51.8	21.8	9.5	0.0	0.0	0.0
**SB-94-T**	94.0	7.1	11.2	43.2	23.3	16.6	5.2	0.4	0.1
**SB-121-T**	121.0	6.8	11.8	43.8	22.5	19.8	2.0	0.0	0.0
**SB-126-T**	126.0	8.8	9.0	40.9	20.9	19.9	9.2	0.0	0.0
**SB-140-T**	140.0	185.5	0.0	1.7	2.0	13.9	33.0	38.6	10.7
**SB-150-T**	150.0	189.4	0.0	1.6	2.0	13.6	32.5	39.1	11.3
**SB-155-T**	155.0	189.8	0.0	1.6	2.0	13.5	32.3	39.3	11.3
**SB-160-T**	160.0	189.8	0.0	1.6	2.0	13.5	32.3	39.3	11.3

Table S6. Mean grain size and percentage of grain size fractions determined through the use of distilled water for the sediment samples of the Daschsbrunnen (DB) core.

Sample ID	Depth (cm)	Mean grain size (µm)	Clay (%)	Fine Silt (%)	Medium Silt (%)	Coarse Silt (%)	Fine Sand (%)	Medium Sand (%)	Coarse Sand (%)
**DB-10-D**	10.0	63.7	0.4	6.9	7.9	29.9	44.1	10.8	0.0
**DB-20-D**	20.0	61.0	0.5	7.2	7.8	31.0	44.5	8.9	0.0
**DB-30-D**	30.0	63.8	0.4	6.5	7.6	31.7	43.9	9.9	0.0
**DB-40-D**	40.0	48.4	0.8	10.8	9.1	32.4	40.3	6.6	0.0
**DB-50-D**	50.0	56.5	0.7	9.5	8.4	30.1	41.6	9.7	0.0
**DB-60-D**	60.0	51.1	0.8	9.7	9.1	33.5	38.5	8.4	0.0
**DB-70-D**	70.0	42.7	0.8	9.5	9.7	39.0	39.5	1.6	0.0
**DB-80-D**	80.0	44.1	0.7	8.4	9.1	40.0	40.7	1.0	0.0
**DB-80-D**	88.0	87.6	0.1	2.5	4.1	28.8	51.7	12.8	0.0
**DB-95-D**	95.0	115.1	0.0	1.9	2.5	18.3	61.5	15.9	0.0
**DB-107-D**	107.0	254.6	0.1	2.0	1.5	5.5	35.0	48.6	7.3
**DB-120-D**	120.0	253.9	0.1	1.9	1.4	5.3	35.9	48.6	6.8
**DB-130-D**	130.0	248.3	0.1	2.0	1.4	5.4	37.5	46.9	6.7
**DB-140-D**	140.0	120.6	0.3	4.4	3.6	11.0	62.6	18.1	0.0
**DB-150-D**	150.0	134.4	0.3	4.2	3.3	9.1	62.7	20.4	0.0
**DB-160-D**	160.0	122.4	0.3	4.3	3.6	10.6	63.1	18.2	0.0

Table S7. Mean grain size and percentage of grain size fractions determined through the use of Calgon for the sediment samples of the Daschsbrunnen (DB) core.

Sample ID	Depth (cm)	Mean grain size (µm)	Clay (%)	Fine Silt (%)	Medium Silt (%)	Coarse Silt (%)	Fine Sand (%)	Medium Sand (%)	Coarse Sand (%)
**DB-10-C**	10.0	5.8	18.7	43.3	17.3	16.2	4.6	0.0	0.0
**DB-20-C**	20.0	4.5	21.2	47.7	18.1	11.9	1.0	0.0	0.0
**DB-30-C**	30.0	4.5	21.3	47.8	18.1	11.8	1.0	0.0	0.0
**DB-40-C**	40.0	4.7	21.0	47.3	16.9	13.8	1.0	0.0	0.0
**DB-50-C**	50.0	4.7	20.9	47.2	16.8	13.7	1.3	0.0	0.0
**DB-60-C**	60.0	4.7	20.9	47.2	16.8	13.7	1.3	0.0	0.0
**DB-70-C**	70.0	4.8	20.6	47.1	16.8	13.7	1.9	0.0	0.0
**DB-80-C**	80.0	8.3	12.4	39.1	16.6	25.4	6.5	0.0	0.0
**DB-80-C**	88.0	8.3	12.3	39.2	16.6	25.4	6.4	0.0	0.0
**DB-95-C**	95.0	73.1	0.3	4.0	4.9	31.1	53.2	6.6	0.0
**DB-107-C**	107.0	75.0	0.3	3.9	4.8	30.2	53.9	7.0	0.0
**DB-120-C**	120.0	256.8	0.2	2.9	2.3	6.8	28.3	49.7	9.8
**DB-130-C**	130.0	253.9	0.2	2.9	2.2	6.8	29.3	49.7	8.8
**DB-140-C**	140.0	110.3	0.3	5.0	4.4	12.4	58.7	19.2	0.0
**DB-150-C**	150.0	108.4	0.3	5.1	4.5	12.7	58.3	19.1	0.0
**DB-160-C**	160.0	113.0	0.3	4.9	4.2	12.1	58.1	20.3	0.1

Table S8. Mean grain size and percentage of grain size fractions determined through the use of Sodium oxalate for the sediment samples of the Daschsbrunnen (DB) core.

Sample ID	Depth (cm)	Mean grain size (μm)	Clay (%)	Fine Silt (%)	Medium Silt (%)	Coarse Silt (%)	Fine Sand (%)	Medium Sand (%)	Coarse Sand (%)
**DB-10-O**	10.0	5.7	16.5	48.6	16.4	14.4	4.1	0.0	0.0
**DB-20-O**	20.0	4.8	20.2	46.9	18.6	13.0	1.3	0.0	0.0
**DB-30-O**	30.0	4.8	20.2	47.2	18.5	12.8	1.3	0.0	0.0
**DB-40-O**	40.0	5.8	15.7	45.8	20.3	16.7	1.4	0.0	0.0
**DB-50-O**	50.0	5.8	15.7	45.8	20.3	16.7	1.5	0.0	0.0
**DB-60-O**	60.0	5.8	15.7	45.8	20.3	16.7	1.5	0.0	0.0
**DB-70-O**	70.0	5.8	15.7	45.8	20.3	16.8	1.4	0.0	0.0
**DB-80-O**	80.0	8.5	12.1	36.7	19.0	27.4	4.8	0.0	0.0
**DB-80-O**	88.0	8.5	12.0	36.6	18.9	27.6	4.9	0.0	0.0
**DB-95-O**	95.0	56.1	0.4	5.0	6.2	39.9	46.3	2.2	0.0
**DB-107-O**	107.0	56.3	0.4	5.0	6.2	39.7	46.6	2.1	0.0
**DB-120-O**	120.0	261.5	2.3	7.8	3.9	7.1	35.0	41.0	3.0
**DB-130-O**	130.0	272.4	2.3	8.0	3.9	7.2	35.4	40.4	2.8
**DB-140-O**	140.0	98.5	0.3	4.7	8.0	22.5	51.8	12.7	0.0
**DB-150-O**	150.0	97.7	0.3	4.7	8.1	22.7	51.3	12.9	0.0
**DB-160-O**	160.0	86.2	0.2	4.4	7.4	21.1	52.0	14.9	0.0

Table S9. Mean grain size and percentage of grain size fractions determined through the use of Sodium pyrophosphate for the sediment samples of the Daschsbrunnen (DB) core.

Sample ID	Depth (cm)	Mean grain size (μm)	Clay (%)	Fine Silt (%)	Medium Silt (%)	Coarse Silt (%)	Fine Sand (%)	Medium Sand (%)	Coarse Sand (%)
**DB-10-P**	10.0	5.7	17.1	46.5	17.6	15.4	3.2	0.2	0.0
**DB-20-P**	20.0	5.2	18.4	46.9	18.9	13.7	2.1	0.0	0.0
**DB-30-P**	30.0	5.1	18.5	47.0	18.9	13.9	1.7	0.0	0.0
**DB-40-P**	40.0	14.2	8.8	26.6	14.5	35.8	14.3	0.0	0.0
**DB-50-P**	50.0	14.2	8.8	26.7	14.6	35.9	14.1	0.0	0.0
**DB-60-P**	60.0	14.2	8.8	26.6	14.5	35.8	14.3	0.0	0.0
**DB-70-P**	70.0	14.0	8.9	26.9	14.6	35.8	13.7	0.0	0.0
**DB-80-P**	80.0	11.3	8.1	28.8	20.9	36.7	5.6	0.0	0.0
**DB-80-P**	88.0	11.4	7.9	28.7	20.9	36.7	5.8	0.0	0.0
**DB-95-P**	95.0	72.3	0.3	4.2	5.6	30.2	52.7	7.1	0.0
**DB-107-P**	107.0	73.9	0.3	4.1	5.5	29.7	52.7	7.7	0.0
**DB-120-P**	120.0	249.1	0.2	3.1	2.3	5.4	34.0	48.4	6.6
**DB-130-P**	130.0	245.2	0.2	3.3	2.4	5.5	34.2	48.4	6.1
**DB-140-P**	140.0	97.1	0.5	6.5	5.2	12.8	58.0	17.1	0.0
**DB-150-P**	150.0	91.9	0.5	6.9	5.5	13.3	58.2	15.6	0.0
**DB-160-P**	160.0	93.8	0.4	6.8	5.4	13.0	58.5	15.9	0.0

Table S10. Mean grain size and percentage of grain size fractions determined through the use of Sodium tripolyphosphate for the sediment samples of the Daschsbrunnen (DB) core.

Sample ID	Depth (cm)	Mean grain size (µm)	Clay (%)	Fine Silt (%)	Medium Silt (%)	Coarse Silt (%)	Fine Sand (%)	Medium Sand (%)	Coarse Sand (%)
**DB-10-T**	10.0	6.2	15.6	45.5	17.7	16.3	4.9	0.0	0.0
**DB-20-T**	20.0	5.6	17.2	46.2	18.7	14.8	3.0	0.0	0.0
**DB-30-T**	30.0	5.5	17.3	46.2	18.7	14.7	3.0	0.0	0.0
**DB-40-T**	40.0	4.7	21.2	46.1	16.8	13.7	2.2	0.0	0.0
**DB-50-T**	50.0	4.7	21.3	46.1	16.8	13.7	2.1	0.0	0.0
**DB-60-T**	60.0	4.7	21.3	46.2	16.8	13.8	1.9	0.0	0.0
**DB-70-T**	70.0	4.7	21.3	46.0	16.7	13.7	2.2	0.0	0.0
**DB-80-T**	80.0	10.2	12.9	29.5	17.2	31.5	8.8	0.1	0.0
**DB-80-T**	88.0	10.3	12.8	29.5	17.2	31.6	8.9	0.1	0.0
**DB-95-T**	95.0	68.5	0.3	4.3	5.3	33.8	50.2	6.2	0.0
**DB-107-T**	107.0	70.3	0.3	4.1	5.2	33.1	50.3	6.9	0.0
**DB-120-T**	120.0	261.5	0.2	2.9	2.3	6.6	27.6	50.1	10.3
**DB-130-T**	130.0	251.6	0.2	3.1	2.4	7.0	28.1	49.0	10.2
**DB-140-T**	140.0	109.4	0.4	5.0	4.3	12.3	59.0	18.9	0.0
**DB-150-T**	150.0	107.4	0.4	5.1	4.5	12.6	59.2	18.3	0.0
**DB-160-T**	160.0	108.5	0.4	5.1	4.4	12.3	59.7	18.1	0.0

Table S11. Mean grain size and percentage of grain size fractions determined through the use of different chemical dispersing agents for IL 1A and IL 1B sediment samples.

Sample ID	Treatment Method	Dispersant	Mean grain size (µm)	Clay (%)	Fine silt (%)	Medium silt (%)	Coarse silt (%)	Fine sand (%)	Medium sand (%)	Coarse sand (%)
**IL-1A-C**	Hydrogen peroxide	Calgon	15.3	9.5	27.0	13.6	29.2	20.7	0.1	0.0
**IL-1A-O**	Hydrogen peroxide	Sodium oxalate	15.4	3.6	30.0	18.4	31.8	15.5	0.6	0.0
**IL-1A-P**	Hydrogen peroxide	Sodium pyrophosphate	17.9	6.2	24.5	14.8	33.8	20.0	0.7	0.0
**IL-1A-T**	Hydrogen peroxide	Sodium tripolyphosphate	19.1	5.6	24.5	15.4	31.1	23.0	0.4	0.0
**IL-1B-C**	Thermal combustion	Calgon	35.8	2.7	12.2	10.0	39.0	33.3	2.8	0.0
**IL-1B-O**	Thermal combustion	Sodium oxalate	30.0	2.8	16.0	13.9	34.2	31.0	2.1	0.0
**IL-1B-P**	Thermal combustion	Sodium pyrophosphate	33.9	2.8	13.5	11.0	36.6	33.0	3.1	0.0
**IL-1B-T**	Thermal combustion	Sodium tripolyphosphate	43.3	2.3	11.2	8.9	33.6	38.6	5.3	0.0

Table S12. Mean grain size and percentage of grain size fractions determined through the use of different chemical dispersing agents for IL 2A and IL 2B sediment samples.

Sample ID	Treatment method	Dispersant	Mean grain size (µm)	Clay (%)	Fine silt (%)	Medium silt (%)	Coarse silt (%)	Fine sand (%)	Medium sand (%)	Coarse sand (%)
**IL-2A-C**	Hydrogen peroxide	Calgon	17.1	6.6	27.8	14.9	27.9	22.4	0.4	0.0
**IL-2A-O**	Hydrogen peroxide	Sodium oxalate	15.5	3.2	29.4	20.3	31.3	15.8	0.0	0.0
**IL-2A-P**	Hydrogen peroxide	Sodium pyrophosphate	17.6	5.9	25.6	16.4	31.2	20.8	0.2	0.0
**IL-2A-T**	Hydrogen peroxide	Sodium tripolyphosphate	19.6	5.5	24.3	15.2	30.8	23.8	0.4	0.0
**IL-2B-C**	Thermal combustion	Calgon	38.0	2.6	13.2	10.0	33.8	35.3	5.1	0.0
**IL-2B-O**	Thermal combustion	Sodium oxalate	31.6	2.3	15.5	14.7	32.6	32.5	2.4	0.0
**IL-2B-P**	Thermal combustion	Sodium pyrophosphate	35.6	2.5	13.8	11.4	33.5	35.5	3.3	0.0
**IL-2B-T**	Thermal combustion	Sodium tripolyphosphate	41.4	2.3	12.2	9.4	32.6	38.6	5.0	0.0
